# Bacteriophage strategies for overcoming host antiviral immunity

**DOI:** 10.3389/fmicb.2023.1211793

**Published:** 2023-06-08

**Authors:** Zhengyu Gao, Yue Feng

**Affiliations:** Beijing Advanced Innovation Center for Soft Matter Science and Engineering, Beijing Key Laboratory of Bioprocess, State Key Laboratory of Chemical Resource Engineering, College of Life Science and Technology, Beijing University of Chemical Technology, Beijing, China

**Keywords:** bacteriophage, prokaryotic immune system, abortive infection, phage-host interactions, anti-immunity measures

## Abstract

Phages and their bacterial hosts together constitute a vast and diverse ecosystem. Facing the infection of phages, prokaryotes have evolved a wide range of antiviral mechanisms, and phages in turn have adopted multiple tactics to circumvent or subvert these mechanisms to survive. An in-depth investigation into the interaction between phages and bacteria not only provides new insight into the ancient coevolutionary conflict between them but also produces precision biotechnological tools based on anti-phage systems. Moreover, a more complete understanding of their interaction is also critical for the phage-based antibacterial measures. Compared to the bacterial antiviral mechanisms, studies into counter-defense strategies adopted by phages have been a little slow, but have also achieved important advances in recent years. In this review, we highlight the numerous intracellular immune systems of bacteria as well as the countermeasures employed by phages, with an emphasis on the bacteriophage strategies in response to host antiviral immunity.

## 1. Introduction

The typical life cycle of bacteriophages (or simply phages) is a complex process that involves various stages, including adsorption, DNA entry, DNA replication, gene transcription and translation, phage assembly, host lysis, and phage burst (Olszak et al., 2017). During the adsorption stage, phages attach themselves to the specific receptor sites present on the surface of bacterial cells. Once bound, DNA entry occurs as the virus injects its genetic material into the host cell. This is followed by DNA replication, transcription and translation of viral genes responsible for enabling the assembly of new phage particles. In the final stages, host lysis and phage burst take place, where the infected host cell is destroyed, resulting in the release of new phages that can go on to infect neighboring cell.

The host deploys a series of immune measures both on the cell membrane and inside the cell to prevent phage infection. The inhibition of adsorption and DNA entry depends on the components of the cell membrane, and the subsequent phage process is mainly countered by the intracellular immune system. Adsorption of phages to the bacterial cell surface is the initial step of infection, which depends on the recognition of specific phage receptors on the cell surface (Labrie et al., 2010). Bacteria have evolved at least three types of blocking mechanisms to prevent phage adsorption, including blocking of phage receptors, production of extracellular matrix, and encoding competitive inhibitors.

After the phage is successfully adsorbed, the injected nucleic acid needs to pass through the outer membrane, peptidoglycan layer, and inner membrane to enter the cell. Notably, some phages do not proliferate immediately after infecting the host bacteria, but integrate their nucleic acids into the host chromosome, replicate during the replication of host nucleic acids, and passage with host cell division. Such phages are called temperate or lysogenic phages, and the phage genomes incorporated into the host chromosome are referred to as prophages (Olszak et al., 2017). Interestingly, these prophages integrated into the host genome may lead to superinfection exclusion, which means a preexisting viral infection prevents a secondary infection with the same or a closely related virus (Folimonova, 2012). Specifically, some prophages can encode membrane-anchored or membrane-component-associated proteins, which prevent other phage DNA from entering host cells, conferring host immunity to the specific phages (Lu and Henning, 1994; Domingo-Calap et al., 2020).

When the phage nucleic acids manage to enter the cell, it is the turn of the intracellular immune system to become the main weapons against the phage. During the past years, restriction-modification systems and CRISPR-Cas systems have been extensively studied as innate and adaptive immune systems respectively, and both have been successfully developed as widely used biotechnology tools (Barrangou and Horvath, 2017; Felice et al., 2019). The great potential for developing biotechnology tools has attracted lots of researchers’ attention to bacterial immune systems. Identifying new prokaryotic defense systems and characterizing their biochemical activities and working mechanisms have become increasingly important research efforts. Interestingly, many antiviral defense genes in bacterial and archaeal genomes show a unique tendency to cluster together to form the so-called “defense islands” (Makarova et al., 2011; Doron et al., 2018). This phenomenon inspired that unknown functional genes around known defense genes in the genome may also participate in the anti-phage defense. Based on this notion, the exploration of genes next to known defense genes led to the discovery of 59 new systems that protect bacteria from phages (Doron et al., 2018; Gao et al., 2020; Millman et al., 2022). In addition, the identification of antiviral systems outside the defense island has also made some progress recently. Results from two groups unveil 31 new defense systems, none of which has been previously detected as enriched in defense islands (Rousset et al., 2022; Vassallo et al., 2022). And some other researches do not specifically emphasize the comprehensive identification of multiple defense systems on a large scale but rather characterize an interesting novel immune system individually (Ofir et al., 2018; Cohen et al., 2019; Xiong et al., 2020; Bernheim et al., 2021; Jaskólska et al., 2022; Johnson et al., 2022; LeRoux et al., 2022; Tal et al., 2022; Zaremba et al., 2022; Zhang et al., 2022). In fact, over 100 novel immune systems have been reported in the past 5 years, highlighting the growing interest in elucidating their functional mechanisms and developing their applications as biological tools.

In turn, phages have also adopted various strategies to antagonize host antiviral immunity. In recent years, significant progress has been made both in the identification of novel prokaryotic immune systems and countermeasures against these defense systems by phages. Here, we review the numerous intracellular immune systems of bacteria as well as the countermeasures employed by phages, with an emphasis on the bacteriophage strategies used to evade these intracellular immune systems. The main strategies will be discussed in two parts, respectively: (1) inactivation of the immune systems by encoding protein inhibitors; (2) bacteriophage gene modifications or mutations.

## 2. Inactivation of the immune system by encoding protein inhibitors

In the case of pathogenic bacteria, effectors refer to proteins that enable the pathogen to modify the host normal cellular processes, thereby promoting the infection process. This pattern is similarly observed in phage-prokaryote interactions, where phage-encoded proteins can inactivate the host immune systems through various mechanisms such as directly binding immune proteins, post-translational modifications of immune proteins, and targeting second messengers (Table 1).

**Table 1:** Summary of the counter-defense strategies using encoding protein inhibitors.

Strategy	Anti-type	Targeting	Mechanism	Reference
Binding immune proteins	Anti-CRISPR proteins	Cascade Cas protein Cas-crRNA	Inhibit the binding or cleavage of nucleic acids, or inhibit the assembly of Cas-crRNA complex	Jia and Patel (2021) and Yin et al. (2023)
	Anti-restriction proteins	REase MTase	Inhibit the cleavage of nucleic acids, stimulate the DNA methylation	Krüger et al. (1983) and King and Murray (1995)
	Anti-RecBCD proteins	RecBCD	Inhibit the cleavage of nucleic acids	Murphy (2000) and Court et al. (2007)
	Anti-TA proteins	Toxin Proteases of antitoxins	Inhibit the toxin, or inhibit the degradation of antitoxin by proteases	Otsuka and Yonesaki (2012)
	Anti-SIR2-containing-system protein	DSR2	Inhibits DSR2 activation	Garb et al. (2022)
	Anti-Gabija protein	GajAB complex	Inhibits the binding and cleavage of nucleic acids	Antine et al. (2023)
PTM of immune proteins	Anti-CRISPR proteins	Csy complex Cas12a	Inhibit the recognition of nucleic acids	Dong et al. (2019) and Niu et al. (2020)
	Anti-TA protein	MazF	Inhibits the toxin	Alawneh et al. (2016)
Targeting second messengers	Anti-CBASS proteins	cGAMP	Cleave or sequester signal molecules	Hobbs et al. (2022), Huiting et al. (2023), and Jenson et al. (2023)
	Anti-Pycsar protein	Cyclic pyrimidine mononucleotide	Cleaves signal molecules	Hobbs et al. (2022)
	Anti-CRISPR protein	Cyclic oligo-adenylate	Cleaves signal molecules	Athukoralage et al. (2020)
	Anti-Thoeris proteins	gcADPR	Sequester signal molecules	Leavitt et al. (2022) and Yirmiya et al. (2023)
Other	Anti-CRISPR protein	DNA	Modifies DNA topology	Forsberg et al. (2021)
	Anti-restriction proteins	DNA SAM	Occlude restriction sites, hydrolysis of SAM	Studier and Movva (1976), Iida et al. (1987), and Krüger et al. (1989)
	Anti-RecBCD proteins	DNA	Protects the injected DNA	Appasani et al. (1999)

### 2.1. Directly binding immune proteins

**Figure 1:**
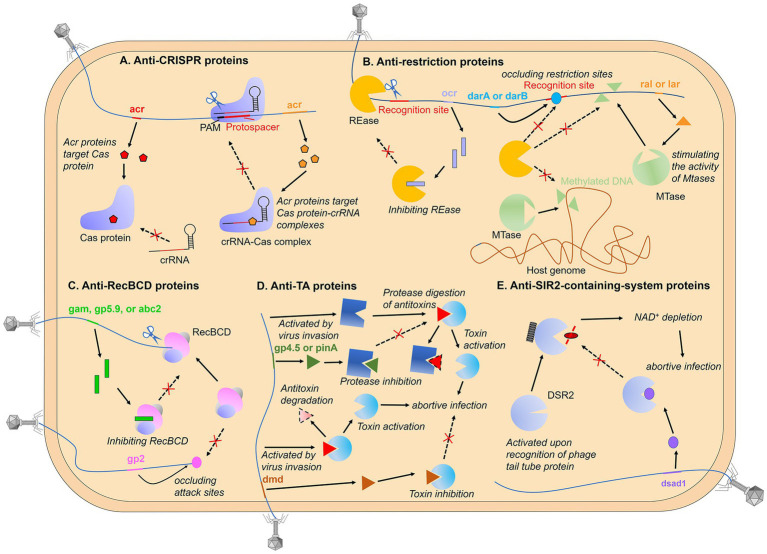
Phage-encoded proteins that direct bind immune proteins. **(A)** the host CRISPR-Cas system targets and cleaves the exogenously invading nucleic acid under the guidance of crRNA upon recognizing the PAM of foreign nucleic acid. Phage-encoded Acr proteins directly target Cas proteins or Cas protein-crRNA complexes, inhibiting recognition or cleavage of phage nucleic acids; **(B)** the restriction-modification (R-M) system is a mechanism that modifies specific sequence motifs in the host genome and degrades unmodified foreign DNA. Phage proteins can circumvent the R-M system through various strategies, such as inhibiting restriction endonucleases (REases), shielding unmodified restriction sites, and stimulating host protein modifications to the phage genome; **(C)** RecBCD degrades DNA lacking Chi and containing free DNA ends, and phage-encoded proteins can directly bind to RecBCD, preventing cleavage of the phage genome, or they can bind to the ends of linear phage genomes to protect the injected DNA from RecBCD attack; **(D)** the antitoxin functions to neutralize the toxin during normal bacterial growth and activate toxins during phage propagation. Phage-encoded proteins can mimic antitoxins or prevent the degradation of antitoxins, thereby avoiding the release of toxins; **(E)** bacterial defense systems with SIR2 domains deplete NAD^+^ upon phage infection, thus triggering abortive infection. Phage-encoded proteins can bind to SIR2-domain proteins and inactivate their enzymatic activity.

*Anti-CRISPR proteins.* Direct binding to host immune protein is the most common inhibition mode among phage protein inhibitors identified at present (Figure 1), which has been widely characterized in the investigation of the anti-CRISPR/Cas mechanisms (Borges et al., 2017). CRISPR-Cas system is an adaptive immune system of prokaryotes, which can obtain foreign nucleic acids as “immune memory” and destroy previously encountered non-self nucleic acid sequences. For more mechanistic information on CRISPR-Cas systems, please see references (Marraffini, 2015; Barrangou and Horvath, 2017). The identification of anti-CRISPR proteins in phages can be traced back to the year 2013 (Bondy-Denomy et al., 2013). Bondy-Denomy et al. found five genes that can inhibit the type I-F CRISPR-Cas system in the prophage sequence of *Pseudomonas aeruginosa* for the first time, called anti-CRISPR genes (Acr genes). Later, the research group found that there was always an Aca (anti-CRISPR-associated) gene downstream of the anti-CRISPR gene, which inspired the identification of numerous Acr genes (Pawluk et al., 2016; Borges et al., 2017). Subsequent structural and biological characterization found that most Acr proteins directly target Cas protein-crRNA complexes, such as Cascade (Yang et al., 2021; Gao Z. et al., 2022; Wang et al., 2022; Yang et al., 2022), Cas9-sgRNA (Dong et al., 2017; Rauch et al., 2017), Cas12-crRNA (Marino et al., 2018; Zhang et al., 2019), Cas13-crRNA (Lin et al., 2020; Meeske et al., 2020), and play a role by shielding the domain of recognition or binding nucleic acid substrate. A small amount of Acr proteins target apo Cas proteins, for instance, AcrIIC2 targets Cas9 and impedes the loading of sgRNA onto Cas9 proteins and the subsequent assembly of complexes (Zhu et al., 2019). Meanwhile, AcrIF3 and AcrIF23 directly inhibit Cas2/3 nucleases, with AcrIF3 mimicking Cas8f-HB (the structural domain responsible for recruiting Cas2/3 nucleases in the Cascade) and binding directly to Cas2/3 to prevent its recruitment (Wang et al., 2016; Sun et al., 2019), while AcrIF23 only suppresses the enzymatic activity of Cas2/3 on DNA and does not prevent its recruitment to the Cascade-dsDNA complex (Ren et al., 2022). And it is noteworthy that the AcrIF5 protein specifically targets Cascade-dsDNA and competes for the binding site of Cas8f-HB, thereby inhibiting Cas2/3 recruitment (Xie et al., 2022). AcrIF5 exhibits a noteworthy complementary relationship with the acrIF3 gene, with all the AcrIF5-encoding genes coexisting upstream of the *acrIF3* gene in the same operon. This complementary functional relationship enables the targeting of Cascade-DNA and Cas2/3, respectively, thereby achieving a thoroughly negative effect on the process of Cas2/3 recruitment by Cascade-DNA.

Moreover, there is a more specific class of Acr proteins, such as AcrIIA1, AcrIIC1, and AcrIIC3, that target Cas9 in all states (free Cas9, Cas9-gRNA, or Cas9-gRNA-DNA). Nonetheless, their distinct mechanisms of action are noteworthy: AcrIIA1 induces Cas9 degradation in a specific intracellular environment (Osuna et al., 2020); AcrIIC1 interacts with the HNH structural domain in Cas9, resulting in the inhibition of substrate cleavage by Cas9, while preserving its recognition and binding to the substrate (Harrington et al., 2017); AcrIIC3 facilitates Cas9 dimerization, which significantly impairs its ability to bind to target DNA and completely eliminates its nuclease cleavage activity (Sun et al., 2019).

*Anti-restriction proteins.* Restriction-modification (R-M) systems exist widely in bacteria and constitute a so-called restriction barrier that prevents interspecies horizontal gene transfer. In these systems, restriction endonucleases (REases) are used to cut invading foreign DNA at specific recognition sites, while the host DNA would not be digested as it is modified at the recognition site by a partner modification enzyme, usually a methyltransferase (MTase; Bickle and Krüger, 1993; Murray, 2002). Protein inhibitors (anti-restriction proteins) are also widespread as anti-R-M strategies. Encoding DNA mimics interacting with the restriction complex can effectively inhibit the activity of R-M systems. A prototypical protein of this type is the coliphage T7 protein Ocr (overcome classical restriction) produced from the gene 0.3. It forms a dimer with each monomer structurally mimicking one helical turn of B-form DNA while the negatively charged surface imitates the distribution of negatively charged phosphate groups along the DNA double helix (Krüger et al., 1983; Walkinshaw et al., 2002). Intriguingly, T7 Ocr is also active against the type I BREX (Bacteriophage Exclusion) system encoded by *Escherichia coli*, a novel defense system that possesses a six-gene cassette consisting of an ATPase-domain protein BrxC, a SAM-dependent N6-adenine methyltransferase BrxX, an alkaline phosphatase domain protein BrxZ, a putative Lon-like protease BrxL, a putative RNA-binding protein BrxB and a small protein BrxA with unknown function (Isaev et al., 2020). Like the R-M system, the BREX system distinguishes self from non-self through methylation of specific motifs in the bacterial genome by BrxX. However, it prevents phage amplification by an unknown mechanism without cleavage of phage DNA. Pull-down assay shows that Ocr binds the BrxX methyltransferase, thus neutralizing the ability of BREX to both methylate and exclude foreign phage DNA (Goldfarb et al., 2015; Gordeeva et al., 2019; Isaev et al., 2020). Similar to Ocr, the coliphage Т4 protein Arn (anti-restriction endonuclease) uses the same principle as an anti-restriction protein (Kim et al., 1997; Ho et al., 2014; Isaev et al., 2020). Not only bacteriophages, but some transmissible plasmids also adopt the same strategy to inhibit R-M systems, which is called Ard (alleviation of restriction of DNA) protein (Zavil'gel'skiĭ et al., 1984; Zavil'gel'skiĭ and Rastorguev, 2009).

Another anti-restriction inhibitor is Stp, an alleviator of DNA restriction encoded by phage T4 that can bind the type I restriction complex *Eco*prrI, inhibiting the restriction but not the modification activity (Amitsur et al., 1989; Penner et al., 1995). Unexpectedly, a bacterial tRNALys-specific anticodon nuclease PrrC is associated with *Eco*prrI and activated by the binding of Stp to EcoprrI, causing the cleavage of tRNA^lys^ and abolishment of protein synthesis (Amitsur et al., 1992). In this way, even if the R-M system is collapsed by phage inhibitor as the first defense line, the PrrC can still provide a secondary line of defense.

Some phages avoid being targeted by the restriction complex through genome modification (see more details below), but bacteria have also acquired the ability to recognize and cleave modified phage DNA in the co-evolutionary arms race. These MDSs (modification-dependent systems) that are contrasted to classical R-M systems, are classified as the type IV R-M system, such as DpnI, Mcr (modified cytosine restriction), Mrr (methylation requiring restriction) and GmrSD (glucose-modified restriction), etc., (Raleigh and Wilson, 1986; de la Campa et al., 1988; Tesfazgi Mebrhatu et al., 2011; Loenen and Raleigh, 2014). Here, the GmrS–GmrD (or GmrSD) system is taken as an example to introduce the battle between phages and bacteria. Two proteins encoded by the GmrSD system form a complex to specifically target and attack glucosylated hydroxymethylcytosine (glc-HMC) modified DNA, in which GmrS protein is responsible for restriction cleavage and GmrD binds to DNA (Bair and Black, 2007). The GmrSD system is inhibited by the T4 phage internal protein I* (IPI*). Immunoprecipitation assays show that IPI* interacts with both GmrD and GmrS, inactivating restriction activity to phage DNA (Bair et al., 2007; Bair and Black, 2007; Rifat et al., 2008). In yet another twist, a single polypeptide produced from a *gmr*SD gene fusion can resist IPI* binding (Rifat et al., 2008).

Finally, it is worth mentioning that not all anti-restriction proteins directly interact with the immune complex, and some phages use indirect, passive strategies to bypass bacterial R-M systems, here we only give a brief introduction. The coliphage P1 proteins DarA (defense against restriction, Dar) and DarB can bind to the phage genome and occlude restriction sites, thus avoiding restriction by the R-M systems (Iida et al., 1987). Modulating cellular methylation processes by phage-encoded effector proteins causes a positive impact on the invasion of viruses. Coliphage λ protein Ral and Lar can alleviate restriction by stimulating the activity of MTases, which allows the phage genome to be rapidly methylated to escape from REases (Zabeau et al., 1980; Loenen and Murray, 1986; King and Murray, 1995). Coliphage T3 encodes an Ado-Met hydrolase that is contributed to overcoming host restriction. As an essential R–M cofactor, S-adenosyl-L-methionine (AdoMet, or SAM) plays a role in both methylation and DNA cleavage reaction (Sistla and Rao, 2004). The hydrolase degrades the methyl donor, preventing DNA methylation and inhibiting SAM-dependent restriction enzymes (Studier and Movva, 1976; Krüger et al., 1989). Recently, a preprint article also demonstrated that this SAMase can also help the host overcome the BREX system through SAM cleavage and inhibition of SAM synthesis (Andriianov et al., 2023).

*Anti-RecBCD proteins.* The linear fragments caused by restriction enzymes acting on foreign DNA will further become substrates for more extensive degradation by the RecBCD, an enzyme cascade complex that contains both helicase and nuclease activities. RecBCD unwinds and digests the blunt-ended DNA until a specific octamer motif that modulates the nuclease activity of RecBCD and results in the subsequent repair of double-strand breaks by homologous recombination, Chi, is encountered. That is, RecBCD degrades DNA lacking Chi and containing free DNA ends, including linear viral DNA present during phage replication or resulting from the action of restriction endonucleases, thus acting as a defense mechanism against phages (Dillingham and Kowalczykowski, 2008; Bobay et al., 2013; Cheng et al., 2020). As a survival guarantee of those phages exposing free DNA ends during the life cycle, anti-RecBCD inhibitors came into being. Phage lambda possesses the gene gam encoding a competitive inhibitor, which mimics the structure of a duplex DNA end to prevent cleavage of the phage genome (Murphy, 1991; Court et al., 2007). Additionally, both phage T7 protein gp5.9 and enterobacteria phage P22 protein Abc2 can directly bind RecBCD to prevent and inactivate it (Poteete et al., 1988; Murphy, 2000; Millman et al., 2020a). Notably, similar to the mechanism of PrrC that is activated only when the first defensive lines have collapsed, RecBCD inhibition by phage protein activates the Retron, an anti-phage defense system comprised of reverse transcriptase (RT) and a non-coding RNA (ncRNA), leading to abortive infection and cell death (Millman et al., 2020a; Bobonis et al., 2022). Besides, in addition to inhibiting RecBCD directly, T4 phage protein gp2 binds the ends of the linear phage genomes and protects the injected DNA from attack by the RecBCD complex (Silverstein and Goldberg, 1976; Appasani et al., 1999).

*Anti-Abi proteins.* The systems described above successfully defend by attacking the nucleic acid of the virus directly, however, bacteria have also developed a defense mechanism called Abi (abortive infection) systems, where infected cells commit suicide or dormancy thus allowing the uninfected bacterial population to survive from phage infection (Lopatina et al., 2020). These Abi systems are usually inactivated to ensure normal bacterial growth and are triggered only when specific substances during phage infection are recognized. Some TA (toxin–antitoxin) systems are well-characterized Abi models, typically, the antitoxin neutralizes the toxin during normal bacterial growth and activates toxins during phage propagation. Toxins are mostly proteins while the corresponding antitoxins are proteins or non-coding RNAs. In addition to directly binding toxins, antitoxins can also inhibit toxin toxicity in an indirect way, such as inhibiting the translation of toxin mRNA or competing for binding to the cellular target, and some antitoxins can regulate transcription of the TA operon with an additional DNA-binding domain. TA systems have been categorized into six types according to the mechanism by which the antitoxin neutralizes the toxin (Schuster and Bertram, 2013; Unterholzner et al., 2013; Chan et al., 2016).

The strategy of phage against TA systems is mainly to make the systems lose their recognized protein targets through gene mutations (to be discussed in detail below). Some phages encode antitoxin mimics that combine with the toxin to re-neutralize the toxin. *P. atrosepticum* Phage ϕTE produces a pseudo-RNA that mimics the antitoxin ToxI of the type III TA system, a repeats-containing RNA that binds and neutralizes the toxic Rnase ToxN (Blower et al., 2012). Phage T4 expresses a protein mimic named Dmd that can replace two antitoxins and bind directly to the RnlA or LsoA toxins (Otsuka and Yonesaki, 2012). Notably, at the position relatively close to dmd homologs in the genomes of T-even phages, a gene gp61.2 encoding anti-DarT protein was identified there. The gp61.2 protein and its homologs help phage evade the DarTG toxin-antitoxin system that ADP-ribosylates phage DNA to disrupt its replication (LeRoux et al., 2022). The phenomenon of anti-defense genes cluster has also been described in the distribution of anti-CRISPR and anti-RM genes, indicating a potential means to identify anti-defense genes (Pinilla-Redondo et al., 2020). Besides, two genes of phage T4 called rIIA and rIIB can help escape a type II TA system, the RexAB system, through unclear mechanisms (Shinedling et al., 1987). Finally, lots of TA systems are protease-dependent, that is, toxin release needs the degradation of antitoxins through proteases (Lehnherr and Yarmolinsky, 1995; Christensen et al., 2001, 2004; Wang et al., 2011), like Lon protease and ClpP protease, suggesting that inactivating these proteases may be an effective anti-TA strategy. This mode of action was demonstrated for phage T7 protein gp4.5 and phage T4 PinA protein (Hilliard et al., 1998; Sberro et al., 2013), which both directly target Lon protease, and for the phage lambda RexB protein, which blocks ClpP protease (Engelberg-Kulka et al., 1998).

*Anti-SIR2-containing-system proteins.* As mentioned above, with the research into “defense islands,” more and more novel prokaryotic immune systems have been identified. Next, we focus on defense systems containing a Sirtuin (SIR2)-domain protein. SIR2 proteins have been characterized as the family of NAD^+^-dependent histone deacetylases in eukaryotes, participating in the regulation of multiple important cellular processes (Imai et al., 2000; North and Verdin, 2004; Schwer and Verdin, 2008). In bacteria, SIR2 proteins have been previously detected as PIWI-associated proteins among the prokaryotic argonautes (pAgos) neighbors (Makarova et al., 2009). As pAgos are involved in nucleic acid-guided cleavage of the phage genome, PIWI-associated SIR2 is presumed to participate in prokaryotic antiviral and confirmed by a recent report (Zaremba et al., 2022). Unsurprisingly, besides pAgo, SIR2-domain proteins were recently shown to be associated with multiple bacterial anti-phage defense systems including Thoeris, AVAST (antiviral ATPases/NTPases of the STAND), DSR (defense-associated sirtuin), and additional unnamed SIR2-containing systems, in which SIR2-domain plays a role of the “effector” in biological conflict, and domains playing similar roles include nucleases, peptidases, phospholipase, etc., (Doron et al., 2018; Burroughs and Aravind, 2020; Gao et al., 2020). Avs (antiviral STAND) proteins have a similar domain architecture to eukaryotic NLR (nucleotide-binding oligomerization domain-like receptors)-related proteins (Platnich and Muruve, 2019), which belongs to STAND (signal transduction ATPases with numerous associated domains) NTPases superfamily (Leipe et al., 2004). Bacterial NLRs have also been described elsewhere (Kibby et al., 2022; Rousset et al., 2022) and these proteins are highly conserved with a central NTPase domain that is flanked by an N-terminal effector and a C-terminal sensor domain. DSR proteins possess an N-terminal SIR2 domain and an uncharacterized C-terminal domain, but lack a central NTPase module, indicating its differences from that of the Avs proteins (Gao et al., 2020). The mechanisms of DSR and AVAST were both described as abortive infection strategies that trigger cell death upon the pattern recognition of phage proteins, such as terminase, portal, or tail tube protein (Garb et al., 2022; Gao L. A. et al., 2022). And it has been confirmed that currently reported various bacterial defense systems with SIR2 domains all deplete NAD^+^ upon infection (Garb et al., 2022). But there is no doubt that phages have also developed resistance strategies against this kind of prokaryotic innate immunity. *B. subtilis* phages phi3T and Spbeta encode genes that inhibit DSR2, called DSAD1 (DSR anti-defense 1), which directly binds to DSR2 competitively with tail tube protein, the activator of DSR2 (Garb et al., 2022). At least three anti-defense proteins can help phages fight against AVAST *in vivo* by an unclear mechanism at present (Gao L. A. et al., 2022). Finally, another SIR2-containing system, Thoeris, and its resistance mechanisms will be discussed below because of its particularity.

### 2.2. Post-translational modification of immune proteins

Post-translational modifications (PTMs) increase the diversity and complexity of proteomes and have vital roles in various cellular processes. In eukaryotic immunity, PTMs of innate sensors and downstream signaling molecules regulate the innate inflammatory response and maintain cellular coordination and balance by inducing their covalent linkage to new functional groups, such as phosphorylation, ubiquitination, SUMOylation, etc. (Liu et al., 2016; Zhou et al., 2017). Protein PTMs are also widespread in bacteria and implicated in all significant physiological processes known to be regulated by PTMs in eukaryotes (Macek et al., 2019). Recently, bacteria have also been reported to use PTMs to regulate the immune response. Millman et al. reported a phage-resistant system encoded by bacteria, which comprises a homolog of ISG15 (Interferon-stimulated gene 15). ISG15 is a ubiquitin-like protein that can be conjugated onto many viruses and host proteins during infection by particular ubiquitin-conjugating enzymes, called ISGylation, like ubiquitination. Notably, a point mutation at the conserved site for ISGylation on the bacterial ISG15-like gene abolished defense, indicating that the ISGylation may also be involved in bacterial immunity (Millman et al., 2022). And about 39% of the CBASS (an Abi immune system present in prokaryotes, see more details below) reported encode Cap2 and Cap3 (CBASS-associated protein 2 and 3) that are homologous to ubiquitin-conjugating (E1/E2) and deconjugating (DUB) enzymes, respectively (Cohen et al., 2019; Millman et al., 2020b). Two groups have reported that E1 catalytic cysteine of the E1-E2 fusion protein Cap2 thioester-linked to the C-terminal glycine of bacterial cGAS that finally leads to the conjugation of the cGAS C-terminus to target proteins in a way similar to ubiquitin transferase-like mechanism. These processes increase the production of cGAMP, which is conducive to resistance to phages. Similarly, the mutation of C-terminal glycine of cGAS eliminated the antiviral activity, highlighting the role of PTMs in CBASS immunity (Jenson et al., 2023; Ledvina et al., 2023).

**Figure 2:**
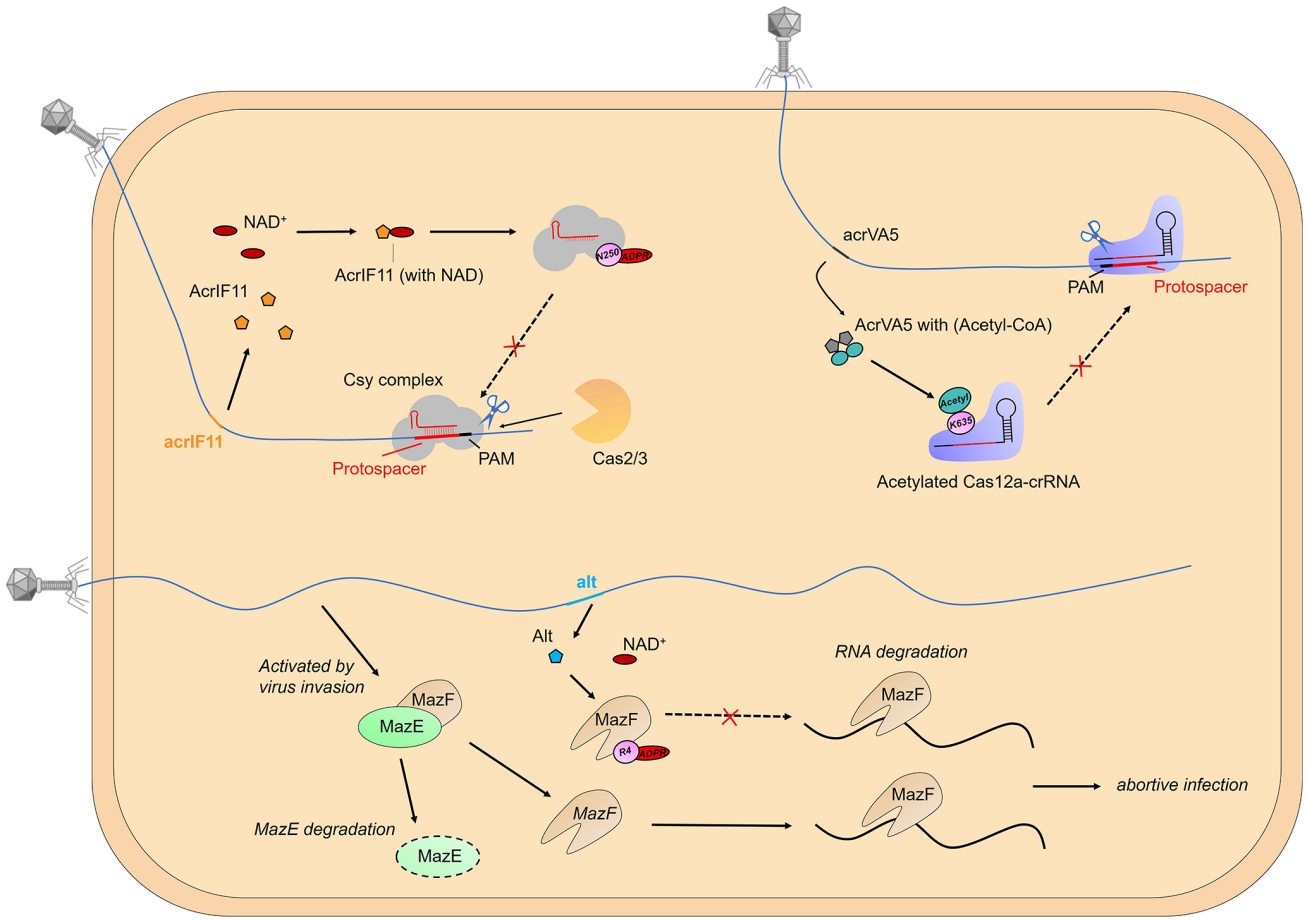
Post-translational modification of immune proteins. Phages have also been found to encode proteins that aid in their invasion of bacterial hosts by modifying bacterial immune proteins through PTMs, mainly observed in the mechanisms of anti-toxin-antitoxin (TA) and anti-CRISPR.

Remarkably, phages have also been found to encode proteins that aid in their invasion of bacterial hosts by modifying bacterial immune proteins through PTMs (Figure 2). This phenomenon has been mainly observed in the mechanisms of anti-toxin-antitoxin (TA) and anti-CRISPR. For example, the phage T4 protein Alt has been described as an ADP-ribosyltransferase that can modify and inactivate the toxin MazF, an RNAase that blocks protein translation and is inhibited by antitoxin MazE (Alawneh et al., 2016). Moreover, biochemical and structural data have revealed that the AcrIF11 protein, encoded by the *P. aeruginosa* prophage, functions as a novel mono-ADP-ribosyltransferase (mART). This mART modifies N250 of the Cas8f subunit located within the Cascade of the type I-F CRISPR-Cas system, a residue that is essential for the recognition of the protospacer-adjacent motif (PAM) (Niu et al., 2020). And a comparable mechanism has been observed in the type V-A CRISPR-Cas system, in which the *Moraxella bovoculi* prophage-encoded AcrVA5 protein acetylates K635 of the Cas12a protein of the V-K CRISPR-Cas system. This residue is also essential for the recognition of the protospacer-adjacent motif, and acetylation results in the complete loss of double-stranded DNA (dsDNA) cleavage activity of Cas12a (Dong et al., 2019).

### 2.3. Targeting secondary messengers

Secondary messengers (or second messengers) are nonprotein molecules or ions that bind to specific target proteins, and disseminate information received by cellular receptors (Newton et al., 2016). In addition to regulating the enzymatic activity of the intracellular metabolic system and controlling the life activities of cells, second messengers are also involved in the antiviral immune process by eukaryotic cells, which use second messengers to transmit viral invasion signals and activate immunity (Wu et al., 2013). For example, the cGAS-STING pathway has a central antiviral effect in the cellular innate immune system. The cGAS (cyclic GMP–AMP synthase) can recognize foreign nucleic acids and utilize GTP and ATP to synthesize cGAMP (cyclic GMP-AMP). As a second messenger, cGAMP binds and activates the endoplasmic reticulum protein STING (stimulator of interferon genes). Through the STING pathway, the signal is transferred to the nucleus, regulates gene transcription, and starts the immune response (Ablasser et al., 2013; Wu et al., 2013; Ablasser and Chen, 2019). Studies into the prokaryotic immune systems show that prokaryotic genes that protect bacteria from bacteriophages have the evolutionary roots of the core components of the eukaryotic innate immune system, including the cGAS-STING pathway and Toll/IL-1 receptor (TIR) domain-containing pathogen receptors, which use second messengers to transmit signals of virus invasion (Whiteley et al., 2019; Ofir et al., 2021; Wein and Sorek, 2022).

Bacterial CD-NTases (the cGAS/DncV-like nucleotidyltransferases) are a large family of phage-defensive oligonucleotide cyclases that share the structural architecture of cGAS but have different product specificities, such as cyclic GMP–AMP, cyclic UMP–AMP, cyclic UMP–UMP, cyclic AMP–AMP–GMP and many more (Cohen et al., 2019; Whiteley et al., 2019; Lau et al., 2020; Ye et al., 2020). These cyclic-oligonucleotide-based anti-phage signaling systems (CBASS) rely on synthesizing cyclic oligonucleotides to activate the abortive infection (Abi) response (Cohen et al., 2019; Lopatina et al., 2020). Effector protein activated by the cyclic oligonucleotides triggers premature cell death through various mechanisms, such as membrane impairment (Cohen et al., 2019), DNA cleavage (Lowey et al., 2020; Fatma et al., 2021), NAD^+^ depletion (Ko et al., 2022; Morehouse et al., 2022), etc. Sacrificing the infected cell deprives the phage of resources for multiplication, thereby protecting against phage infection. Such systems are found in about 13% of prokaryotic genomes and are present in all major bacterial phyla as well as in archaea (Whiteley et al., 2019; Millman et al., 2020b). Diverse operon organization, signaling molecules, and effector function of CBASS classify them into four major types (type I–type IV) (Millman et al., 2020b). Recent studies have shown that in addition to cyclic-oligonucleotide, cyclic pyrimidines are also used as a second messenger to transmit signals in prokaryotic antiviral immunity, which has not been reported in eukaryotic cells. Pyrimidine cyclase systems for anti-phage resistance, called Pycsar, are widespread in prokaryotes and work in similar ways as the CBASS (Tal et al., 2021).

The Toll/Interleukin-1 receptor (TIR) domain is a canonical component of animal and plant immune systems (Fitzgerald and Kagan, 2020). Bacteria have also been found to encode TIR domain-containing proteins, and subsequent studies have shown that the generation of intracellular signaling molecules is a conservative function of the TIR domain in both plant and bacterial immunity (Wan et al., 2019; Ofir et al., 2021; Yu et al., 2022). A representative example is the bacterial Thoeris system, an anti-phage defense system composed of ThsA and ThsB proteins. Phage infection triggers TIR domain-containing protein ThsB to produce cyclic ADP ribose isomer, a second messenger binding to the SIR2 NADase domain-containing protein ThsA specifically and resulting in the activation of NADase activity, which then depletes the cells of the essential molecule nicotinamide adenine dinucleotide (NAD^+^), leading to abortion infection and cell death (Ofir et al., 2021).

**Figure 3:**
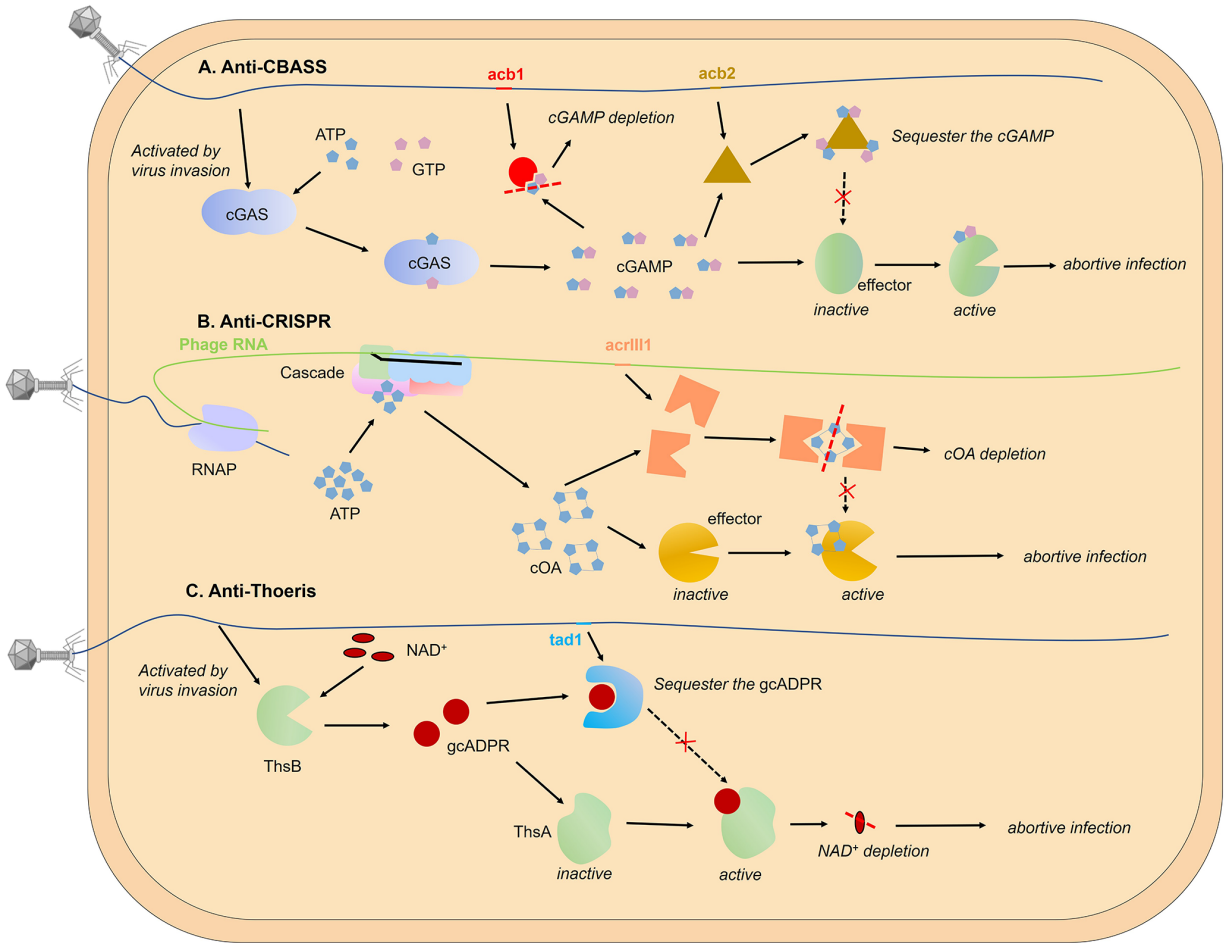
Targeting secondary messenger. The use of second messengers to signal viral invasion has been reported in various prokaryotic systems, including CBASS **(A)**, CRISPR-Cas system **(B)**, and Thoeris system **(C)**. Phage-encoded proteins can degrade second messengers in an enzymatically active form or bind and sequester them in a non-enzymatically active form without cleaving small molecules. Pyrimidine cyclase systems, referred to as Pycsar, exhibit analogous functionality to the CBASS mechanism. The anti-Pycsar protein Apyc1 and anti-CBASS protein Acb1 act as inhibitors of anti-phage defense through the cleavage of second messengers, demonstrating a similar mechanism. For reference, please refer to Figure 3A for the mechanism models of both Pycsar and Apyc1.

It seems that phages can always find counter-strategies against bacterial immunity, with no exception for CBASS, Pycsar, and Thoeris (Figure 3).

*Cleave signal molecules*. Hobbs et al. have reported the first anti-CBASS protein Acb1 and anti-Pycsar protein Apyc1 that degrade the cyclic nucleotide messengers to inhibit anti-phage defense (Hobbs et al., 2022). Structural analyses of Acb1 in complex with 3′,3′-cGAMP reveal the mechanism of metal-independent hydrolysis 3′ of adenosine bases that allows broad recognition and degradation of cyclic dinucleotide and trinucleotide signals. Crystal structures of Apyc1 define a metal-dependent cNMP phosphodiesterase, which targets and hydrolyzes a wide range of cyclic pyrimidine mononucleotide signals with relaxed specificity. In fact, the arms race between bacteria and phage around the second messenger has long been found in the type III CRISPR-Cas system. The coupling with the abortive infection mechanism defines the specificity of the type III CRISPR/Cas system. After recognizing the target RNA strand, this system not only shows the ability to cut the nucleic acid substrate but also activates the synthesis of cyclic oligo-adenylate (cA_n_) second messengers, an activator of downstream effectors that results in cell death and prevents viral propagation (Molina et al., 2020; Kolesnik et al., 2021). Athukoralage et al. identified a type III anti-CRISPR protein (AcrIII1) that rapidly degrades cyclic tetra-adenylate (cA_4_). Crystal structure of AcrIII1 in complex with cA_4_ show that a molecule of cA_4_ bound at the dimer interface of two AcrIII1 and is cleaved by a conserved active site (Athukoralage et al., 2020).

*Sequester the second messenger.* Besides cleaving signal molecules, there is a class of phage proteins that bind and sequester the second messenger without enzymatic activity. This kind of protein was first found in the gene that helps phages escape from the Thoeris system, called Tad1 (Thoeris anti-defence 1). Tad1 protein shows broad-spectrum inhibition as it can sequester molecules derived from a TIR-domain protein of both plants and bacteria (Leavitt et al., 2022). A similar mechanism has been found in the anti-CBASS mechanism, Huiting et al. revealed that the Acb2 protein encoded by *P. aeruginosa* phage displays a high binding affinity towards 3′, 3’-cGAMP molecules, but lacks enzymatic activity (Huiting et al., 2023). Through structural analyses of the Acb2 protein and its complexes with 3′, 3’-cGAMP, and c-di-AMP, it was determined that the Acb2 protein exists as a compact hexameric structure, with each 3′, 3’-cGAMP molecule residing within a “binding pocket” formed by the N-terminal ends of two Acb2 monomers, thereby facilitating stable interactions. By selectively adsorbing and isolating 3′, 3’-cGAMP molecules, Acb2 effectively disrupts the immune action of the CBASS encoded by *P. aeruginosa*. Furthermore, Acb2 has also been discovered and characterized in coliphage T4 (called Vs.4 in T4 phage), exhibiting a similar binding mechanism (Jenson et al., 2023).

Lastly, of the various immunosuppressive agents discussed above, anti-CRISPR proteins exhibit the greatest diversity in terms of number, variety, and mechanism of action. This can be attributed to the stringent regulation by the aca proteins, which significantly facilitates the identification and characterization of anti-CRISPR proteins. However, not all anti-defense genes have such trans-acting factors downstream. In numerous instances, anti-defense genes are discovered by comparing genomic variations between host-sensitive and non-sensitive phages. Typically, host-insensitive phages are repelled by the host immune system, whereas host-sensitive phages are more likely to have evaded immunity by encoding antagonistic proteins, enabling successful infection of the host. In this way, in a recent preprint article, Yirmiya et al. identified suppressor proteins of three novel bacterial immune systems, namely Gabija, Thoeris and Hachiman (Antine et al., 2023; Yirmiya et al., 2023). Specifically, Gad1 (Gabija anti-defense 1) binds the Gabija complex as an octamer and impedes its ability to bind and cleave DNA. Tad2 (Thoeris anti-defense 2) exhibits a mechanism similar to Tad1, inhibiting Thoeris defense by binding and sequestering bacterial immune signaling molecules, thereby preventing the activation of the Thoeris immune effector. While Had1 (Hachiman anti-defense 1) was identified, its functional mechanism remains unknown.

## 3. Genes modifications or mutations that help phages evade the immune system

### 3.1. Mutations or modifications of non-coding sequences

**Figure 4:**
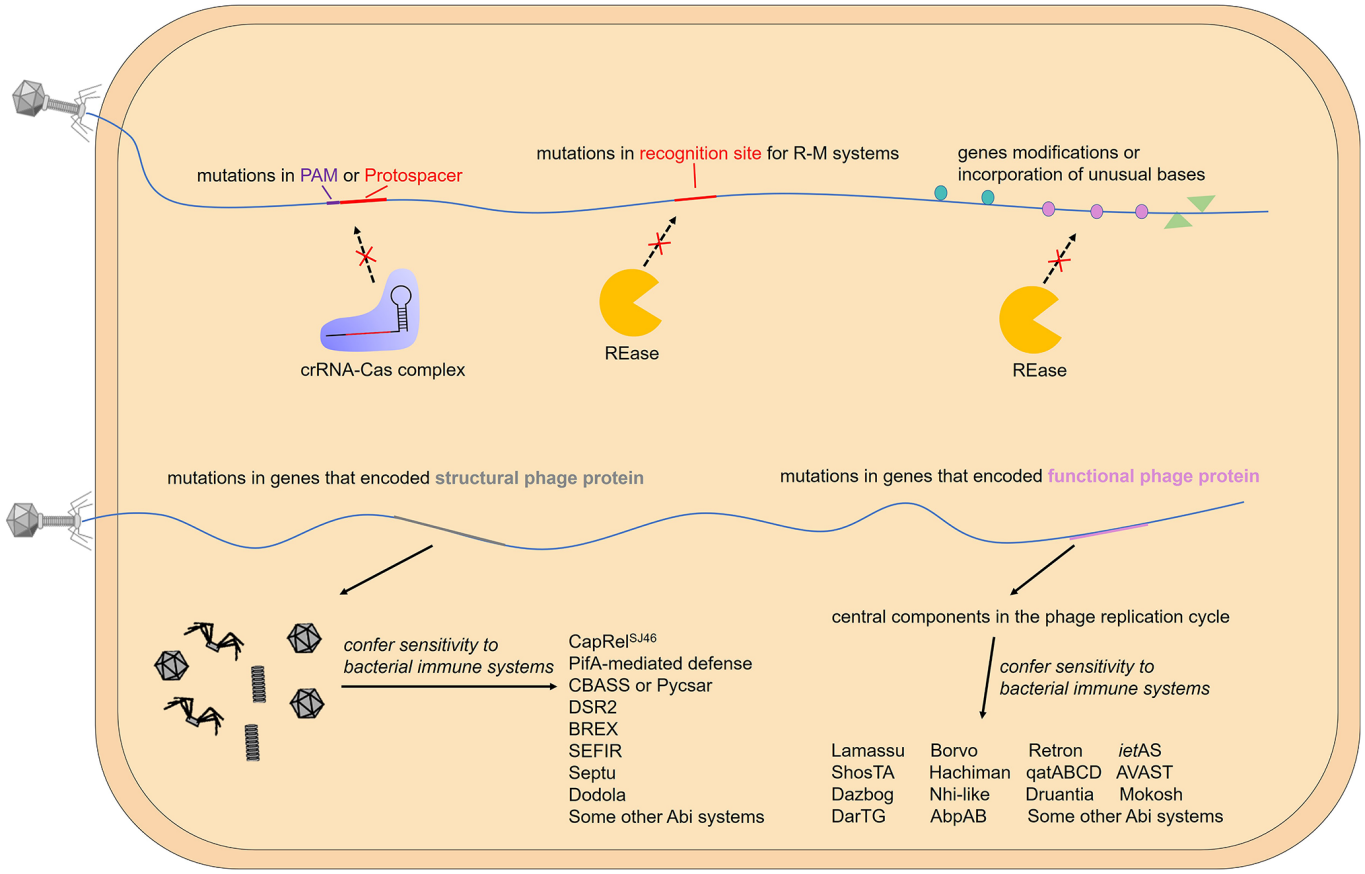
Gene modifications or mutations that help phages evade the immune system. The activation of some bacterial immune systems relies on the recognition of specific phage motifs, while others rely on the recognition of specific phage proteins. When specific motifs are targeted by the host, mutations, modifications, and base substitutions can effectively help the phage evade immunity. On the other hand, when the expressed protein is targeted, the phage usually mutates the gene encoding it.

Differences in genome modifications between a host and its phage allow self from non-self-discrimination, which means that the same DNA modification as the host may help phages overcome the restriction–modification systems and related defense, such as the BREX system (Gordeeva et al., 2019). In fact, phage DNA has been found to become methylated by methyltransferases from the host or phages themselves. For example, certain phage proteins mentioned earlier can stimulate the activity of host MTases, thereby facilitating prompt methylation of the phage genome. However, upon infecting methyltransferase-deficient bacteria, newly-formed virions will be unmethylated and once again become phenotypically sensitive to REases. In order to enhance their chances of survival, some phages have acquired a competitive edge by integrating a cognate orphan MTase, which allows them to encode their own MTases. This phenomenon is observed in various phages such as *B. subtilis* phage SPR and *L. lactis* phage φ50 (Günthert and Reiners, 1987; Hill et al., 1991; Murphy et al., 2013). Notably, another RM-like system recently identified, called DISARM (defense island associated with restriction–modification), still protected against the phages propagated from drmMII-expressing strain, in which genome has been hypermethylation by DrmMII, a C-5 cytosine-specific DNA methyltransferase of DISARM system (Ofir et al., 2018). These results indicate an unusual mechanism to identify invading phage DNA that differs from classic R-M systems. In addition to the same methylation modification as the host DNA, other modifications and incorporation of unusual bases can also protect the phage genome from cleavage by the host REases (Figure 4). One example is the gene mom of coliphage Mu, which encodes an enzyme converting adenine to N6-(1-acetamido)-adenine in the phage genome (Hattman, 1980; Drozdz et al., 2012). Moreover, the genome of several *B. subtilis* phages possess the unusual base hydroxymethyl uracil that replaces thymine, and coliphage T4 contains hydroxymethyl cytosine (HMC) or glucosylated HMC (glc-HMC) instead of cytosines to make these sites unrecognizable and thus circumvent the R-M, and even some CRISPR-Cas mechanisms (Warren, 1980; Weigele and Raleigh, 2016; Vlot et al., 2018; Table 2).

**Table 2:** Summary of the counter-defense strategies by genes modifications or mutations.

Strategy	Position/type	Bypassed system	Reference
Genes mutations	PAM or protospacer	CRISPR-Cas systems	Deveau et al. (2008), Sapranauskas et al. (2011), and Semenova et al. (2011)
	Restriction recognition sites	R-M systems	Krüger et al. (1995) and Pleška and Guet (2017)
	Structural phage proteins (capsids, tail tube, tail fiber, head fiber, etc.)	CapRel^SJ46^ PifA-mediated defense CBASS or Pycsar DSR2 BREX SEFIR Septu Dodola Some other Abi systems	Champness and Snyder (1982), Molineux et al. (1989), Schmitt et al. (1991), Bingham et al. (2000), Labrie et al. (2012), Tal et al. (2021), Garb et al. (2022), Zhang et al. (2022), Huiting et al. (2023), and Stokar-Avihail et al. (2023)
	Functional phage proteins (DNA polymerase, ssDNA binding protein, helicase, terminase, etc.)	DarTG system dNTP-depletion-mediated defense AbpAB Lamassu Borvo Retron ietAS ShosTA Hachiman qatABCD AVAST Dazbog Nhi-like Druantia Mokosh Some other Abi systems	Bouchard and Moineau (2004), Bidnenko et al. (2009), Haaber et al. (2009), Haaber et al. (2010), Samson et al. (2013a), Yasui et al. (2014), Chen et al. (2017), Montgomery et al. (2019), LeRoux et al. (2022), Tal et al. (2022), and Stokar-Avihail et al. (2023)
Genes modifications	Methylation	R-M systems BREX system	Loenen and Murray (1986), Labrie et al. (2010), Murphy et al. (2013), and Gordeeva et al. (2019)
	Glucosylation	CRISPR-Cas systems BREX system	Vlot et al. (2018) and Gordeeva et al. (2019)
	Other modified bases	R-M systems	Warren (1980) and Weigele and Raleigh (2016)

Different R-M systems have unique preferences for the recognition motifs of viral DNA, and the antiviral efficiency is influenced by the number, and the orientation of the specific recognition motif and even the distance between them (Meisel et al., 1992; Krüger et al., 1995; Pleška and Guet, 2017). These factors result in a selective advantage for those phages whose restriction recognition sites in their genomes are congenital insufficiency or underrepresented via the accumulation of point mutations. This strategy is also used by phages against CRISPR-Cas systems. The guide crRNA comes from the transcription and processing of the CRISPR array, in which the spacers separated by the repeats come from the adaptive acquisition of foreign nucleic acid, while the protospacer on the genome of bacteriophages or other MGEs (mobile genetic elements) does not have a bacterial conserved repeat region next to it. Therefore, CRISPR-Cas system distinguishes self from non-self through the recognition of PAM (Marraffini and Sontheimer, 2010). For phages, in addition to encoding Acr proteins, mutations in protospacer or PAM can also help them escape the recognition and targeting of the bacterial CRISPR-Cas system (Deveau et al., 2008). Notably, RNA-guided Cas nuclease can cut DNA sequences that do not match the spacer incompletely, which causes off-target effects (Fu et al., 2013). And a requirement for a complete pairing between the spacer on crRNA with the sequence close to PAM on the protospacer called “seed regions” is essential for target strand binding, which means substitutions in the protospacer region that are close to PAM can more effectively avoid targeting of CRISPR-Cas system (Sapranauskas et al., 2011; Semenova et al., 2011). Interestingly, AcrIIA22 and its homologs, discovered in clostridial bacteria and their prophage, are reported to function as a DNA nickase that can relieve the torsional stress of DNA and thus convert it into a Cas9-resistant conformation (Forsberg et al., 2021), as the torsional stress in DNA modulates the formation of R-loop and further efficiency of Cas9 cleavage (Szczelkun et al., 2014; Farasat and Salis, 2016; Ivanov et al., 2020). This indicates that modifying DNA topology, like DNA modification or mutation, is also an effective way for MGEs to escape bacterial CRISPR-Cas system.

Recently, a novel defense system involving SMC proteins called Lamassu is reported to protect against phage via abortive infection (Doron et al., 2018; Millman et al., 2022). While Lamassu systems were originally speculated to be activated by recognizing phage DNA replication intermediates (Jaskólska et al., 2022; Millman et al., 2022), the actual activator is more like to be protein–DNA complexes that are produced uniquely by either palindromic sequences or certain types of DNA damage (Robins et al., 2022). As the palindromic DNA sequences can trigger Lamassu-dependent cell death, introducing mutations artificially in palindromic sequence can avoid Lamassu sensing, suggesting that mobile genetic elements such as phages may also be able to bypass the Lamassu system by mutations.

### 3.2. Mutations of coding sequences

The strategies mentioned above are based on the fact that foreign nucleic acids activate the immune system. In other cases, similar to pathogen-associated molecular patterns (PAMPs) recognized by eukaryotic innate immune systems (Medzhitov, 2001), some specific phage proteins are utilized by bacteria to sense viral invasion. Therefore, mutations or deletions of genes encoding these proteins can effectively help bacteriophages escape from bacterial immunity (Figure 4). A typical example is phage capsid protein, and multiple prokaryotic immune systems are triggered by this unique type of structural phage protein. Viral capsids are the protein shell in a shape of a polygon-like sphere or a helix that encases the nucleic acid. Targeted by a variety of defense systems, the huge evolutionary pressure results in inevitable mutations of capsid-coding genes. To our knowledge, T4 phages carrying mutations in gp23 (encode peptide Gol that derived from T4 capsid) escape the Lit Abi system (Champness and Snyder, 1982; Bingham et al., 2000); Phage SECΦ27 capsid protein gp57 triggers a TA system named CapRel^SJ46^ and mutations in the corresponding gene help escape (Zhang et al., 2022); Simultaneous mutations in gp1.2 and gp10 (T7 capsid protein) allow phage T7 to escape the PifA-mediated defense (Molineux et al., 1989; Schmitt et al., 1991); Phages PaMx41and T5 can escape CBASS and Pycsar, respectively, through mutations in the major capsid gene but there is no direct evidence that capsid activates CBASS or Pycsar (Tal et al., 2021; Huiting et al., 2023); Comparative genomic analyses of AbiT-insensitive phages showed that mutations in the major capsid protein or other two early-expressed phage genes help *L. lactis* virulent phages evade AbiT, an Abi system which molecular mechanism remains unknown (Labrie et al., 2012).

In addition to structural phage proteins, some functional phage proteins expressed in the cell during infection are also sensed by defense systems, and these proteins are related to intracellular processes including DNA replication, recombination, repair, and host transcription shut-off, etc. For example, in the DarTG TA system mentioned above, mutations on gene mga42 encoding a DNA polymerase and an unknown functional gene mga32 allow SECϕ18 phages to escape DarTG-mediated defense (LeRoux et al., 2022). Genome analysis of phage mutants that evade certain defense systems shows that the gene involved in sensitivity to the AbiK system (sak gene) is related to single-strand annealing proteins involved in homologous recombination or DNA repair (Bouchard and Moineau, 2004); genes involved in sensitivity to AbiQ system (saq gene) also have been proven to participate in DNA replication, repair or recombination (Samson et al., 2013a); mutations in gene 41 of phage T4 that avoid AbpAB system, encodes a replicative DNA helicase that plays an essential role for DNA replication (Yasui et al., 2014). In addition, genes involved in sensitivity to AbiV and AbiD, and some other Abi systems, have been located on the phage genome, but their function is unknown (Bidnenko et al., 2009; Haaber et al., 2009, 2010; Chen et al., 2017; Montgomery et al., 2019).

Interestingly, phage proteins with counter-defense functions can also be sensed by the host immune system. For example, the Ocr protein, a phage-encoded protein inhibitor that can inactivate the BREX and R-M systems, has recently been shown to as a trigger for multiple defense systems, including PARIS, Gabija, and Zorya type II (Rousset et al., 2022; Wu et al., 2022). This clever strategy, similar to PrrC and Retrons, can activate additional anti-phage mechanisms when the first line of defense fails. Mutations in the ocr gene could help phage evade these systems (Wu et al., 2022), but also re-expose phage to immune threats that could otherwise be overcome by the Ocr protein.

Modulating host RNA polymerase is required to make it serve viral needs, and covalent modifications or RNA polymerase-binding proteins result in host transcription shut-off, which would otherwise hamper phage multiplication (Nechaev and Severinov, 2003). Some defense systems can sense phage-mediated inhibition of host transcription. For example, host gene expression is immediately shut off caused by the invasion of phage T4, leading to an insufficient supply of antitoxin RnlB which then triggers the consequent activation of toxin RnlA (Svenson and Karlström, 1976; Koga et al., 2011; Otsuka and Yonesaki, 2012). Recently, a novel defense system has been identified, which can block phage replication by depleting deoxynucleotides, and is also considered to be activated by sensing transcriptional inhibition. The reason is that mutation of a gene gp5.7 in the phage T7 that is responsible for shutting down RpoS-dependent RNAP transcription, despite slight growth defects, is not interfered with by the dNTP-depletion-mediated defense (Tabib-Salazar et al., 2018; Tal et al., 2022). Very recently, Rotem Sorek’s group studied the activation factors of more than 50 defense systems by analyzing phage escape mutants, almost including all the newly identified prokaryotic immune systems. This study proposed that central components in the phage replication cycle commonly confer sensitivity to bacterial defense systems, which greatly promoted the decoding of the activation mode of the bacterial defense system (Stokar-Avihail et al., 2023).

## 4. Conclusion

This review summarizes prokaryotic anti-phage measures and corresponding counter-defense strategies around a process described as an ‘arms race’ between bacteria and their bacteriophages. Research in this field used to focus on R-M, CRISPR-Cas, and some Abi mechanisms. Many excellent reviews have covered this topic in depth (Labrie et al., 2010; Samson et al., 2013b; Hampton et al., 2020). However, since 2018, researches into defense islands and revenge of the phages have been keeping rapid growth (Doron et al., 2018; Gao et al., 2020; Millman et al., 2022; Tal and Sorek, 2022), which inspired our expanded discussion on new discoveries over past few years. These studies have brought at least two important potential values. First, fundamental research into interactions between phages and bacterial hosts expanded biotechnology tools and helped evolve and screen phages or strains with designated resistance, which have underpinned the development of many fields, including but not limited to gene editing (Adli, 2018; Manghwar et al., 2019), clinical therapy (Abedon, 2019; Dedrick et al., 2019), food industry, and agriculture (Nakai and Park, 2002; O'Sullivan et al., 2019). Secondly, another exciting discovery is that a variety of defense proteins of the human innate immune system have direct homologs in bacteria (Wein and Sorek, 2022). Evolutionary conservatism has brought some functional similarities, enabling researchers to decipher the eukaryotic immune mechanism in a relatively simple bacteria-phage experimental model and reveal unprecedented potential therapeutic targets.

One area that requires substantial attention is how bacterial defense systems sense phage infection. Characterization of the activation mode of the immune system lags far behind the identification of new immune systems. Understanding how the immune systems sense invasive MGEs is of great significance for phage-based antibacterial treatments, which will help to genetically modify phages and help them bypass the immune recognition of pathogens. Moreover, most of the studies on defense systems have rarely or limited consideration of other co-existing anti-phage mechanisms. A variety of defense systems are clustered in the defense islands and provide abundant resistance against phages. The priority of their activation must be strictly regulated and the conditions for bacteria to prefer different resistance mechanisms are not fully studied. For example, the systems that respond to the invasion through the Abi mechanism make biological sense only when the phage reaches a stage as late as possible of its infection cycle or when other mechanisms are insufficient to deal with the threat, just like PrrC and Retrons (Amitsur et al., 1992; Bobonis et al., 2022). How do the first lines of defense, non-Abi systems work synergistically in the same bacterial cell (Dupuis et al., 2013; Silas et al., 2017)? The effects of conjoint resistance mechanisms on phage population and evolution have rarely been assessed. Furthermore, beyond the individual range, from the perspective of bacteria and bacteriophage communities, the cooperation between individuals should be further studied (Borges et al., 2018; Bernheim and Sorek, 2020), which will further promote the more accurate use of microbial resources.

Finally, more researches should be conducted on ‘anti-defense islands’ (Pinilla-Redondo et al., 2020; LeRoux et al., 2022). Genes without functional annotations but clustered next to genes with clear anti-defense functions may also encode anti-defense proteins. These unknown genes within these regions may provide a wide range of regulatory tools to help us better tame the bacterial defense systems.

## Author contributions

ZG: writing – original draft. YF: writing – review and editing. All authors contributed to the article and approved the submitted version.

## Funding

This work was supported by National Key Research and Development Program of China (2022YFC3401500 and 2022YFC2104800) to YF. Beijing Nova Program (20220484160), the Fundamental Research Funds for the Central Universities (QNTD2023-01).

## Conflict of interest

The authors declare that the research was conducted in the absence of any commercial or financial relationships that could be construed as a potential conflict of interest.

## Publisher’s note

All claims expressed in this article are solely those of the authors and do not necessarily represent those of their affiliated organizations, or those of the publisher, the editors and the reviewers. Any product that may be evaluated in this article, or claim that may be made by its manufacturer, is not guaranteed or endorsed by the publisher.
